# The relict plant *Tetraena mongolica* plantations increase the nutrition and microbial diversity in desert soil

**DOI:** 10.3389/fpls.2025.1539336

**Published:** 2025-03-20

**Authors:** Yanan Quan, Xiuwen Gan, Shiyun Lu, Xiaodong Shi, Mingsheng Bai, Yin Lin, Yufei Gou, Hong Zhang, Xinyue Zhang, Jiayuan Wei, Tianyu Chang, Jingyu Li, Jianli Liu

**Affiliations:** ^1^ College of Biological Science and Engineering, North Minzu University, Yinchuan, Ningxia, China; ^2^ Key Laboratory of Ecological Protection of Agro-pastoral Ecotones in the Yellow River Basin, National Ethnic Affairs Commission of the People’s Republic of China, Yinchuan, Ningxia, China; ^3^ Ningxia Key Laboratory of Microbial Resources Development and Applications in Special Environment, Science and Technology Department of Ningxia, Yinchuan, Ningxia, China

**Keywords:** *Tetraena mongolica*, soil nutrition, bacterial communities, fungi communities, root zone soil, bare soil

## Abstract

**Introduction:**

*Tetraena mongolica *was established in the West Ordos Region of northwest China approximately 140 million years ago. It plays an irreplaceable role in maintaining local ecosystem stability.

**Methods:**

This study aimed to evaluate the effects of planting *T. mongolica* on soil nutrition and microbial communities by comparing the root zone soil (Rz_soil) and bare soil (B_soil) across three different plant communitie.

**Results:**

The results showed that *T. mongolica* decreased soil pH and Na^+^ while increasing available potassium, soil organic matter, organic carbon, total nitrogen, and potassium. *T. mongolica* significantly improved the diversity indices (Sobs and Ace), as well as the richness index (Chao), of bacterial and fungal communities across three plant communities. Meanwhile, the relative abundances of *Rubrobacter* and norank_c_Actinobacteria in the bacterial communities declined significantly in the Rz_soil compared with the B_soil across all three plant communities. In contrast, the relative abundances of *Fusarium* and *Penicillium* were higher, whereas those of *Monosporascus* and *Darksidea* were lower in Rz_soil than in B_soil in the two plant communities. *T. mongolica *decreased the soil bacterial co-occurrence networks while increasing the soil fungal co-occurrence networks.

**Discussion:**

These results provide a new perspective to understand the role of *T. Mongolica* in the desert ecosystems.

## Introduction

Soil is the foundation of terrestrial ecosystems, and serves as a crucial site for material cycling and energy conversion between biotic and abiotic environments (Lehmann et al., 2020; Creamer et al., 2022). It is a multicomponent complex characterized by intricate physical, chemical, and biological properties, providing essential growth medium and conditions, such as nutrients, water, and air, which are necessary to support plant life (Zhang et al., 2021; Hartmann and Six, 2023). Soil properties are influenced by various environmental factors including temperature, moisture, oxygen levels, and organisms (Crowther et al., 2019; Zheng et al., 2019; Patel et al., 2021; Feng et al., 2022). Plants are the most significant biological factors affecting soil structure and characteristics (de Vries et al., 2023). Plants influence several soil properties, including organic matter transformation, water cycling, and community composition through mechanisms, such as root exudates, litter decomposition, and regulation of the field microclimate. In this interaction, plants first alter soil properties and, in turn, affect plant growth, known as “plant-soil feedback” (Beckman et al., 2023; de Vries et al., 2023; Ali et al., 2024; Zou et al., 2024). Microorganisms are an important component of soil (Fierer, 2017) and are also involved in plant-soil feedback processes (Saleem et al., 2019; Semchenko et al., 2022). Microorganisms play crucial roles in soil nutrient cycling (Zhang et al., 2021). On the one hand, microorganisms can convert soil nutrients into forms that plants can utilize (Philippot et al., 2024). On the other hand, they also can decompose and mineralize plant litter and root exudates into soil components (Chen et al., 2021; Coban et al., 2022). Therefore, soil microorganisms play a bridging role in “plant-soil feedback.”

The West Ordos Region of Inner Mongolia, located in Northwest China on the eastern edge of the Asian desert, features a natural geographical landscape dominated by steppification deserts. The region is arid and experiences minimal rainfall, a significant water deficit, soil infertility, and a lack of essential nutrients. The flora primarily consists of xerophytes, superxerophytes, salt-tolerant shrubs, and semi-shrubs (Guo et al., 2024b). The West Ordos Region is also known as the “refuge” of ancient Mediterranean relict plants. Because of edge effects and paleogeography, most keystone species and dominant species of the plant community in the West Ordos region are ancient Mediterranean relict species (Li et al., 2018). *Tetraena mongolica* is a strong xerophytic succulent leafy deciduous shrub that belongs to the Family Zygophyllaceae. This species is a unique example of an ancient relict plant in China, often referred to as the “living fossil”. It is classified as a nationally endangered and rare protected species and represents one of the characteristic genera of the Mongolian Plateau and Central Asia (Cheng et al., 2020; Liu et al., 2023a). *T. mongolica* is exclusively found in western Etuoke County, Wuhai City, within the Inner Mongolia Autonomous Region, and in Shizuishan City of the Ningxia Hui Autonomous Region. As a keystone species in desert ecosystems, *T. mongolica* plays an irreplaceable role in maintaining the local ecosystem stability and protecting the ecological environment (Liu et al., 2023b).

Currently, research on *T. mongolica* has primarily focused on the physical geography of its distribution (Guo et al., 2024a), biological characteristics, eco-physiological adaptations, chemical composition (Wu et al., 2020), and genetic structure (Cheng et al., 2020; Dang et al., 2020). However, a systematic analysis of the local soil altered by *T. mongolica* is lacking. This study aimed to assess the effects of *T. mongolica* cultivation on soil nutrition and microbial communities. These findings provide a new perspective to understand the role of *T. Mongolica* in the desert ecosystems.

## Materials and methods

### Study site

The study area is situated in the Gander Mountain Core Protection Area of *T. mongolica*, located in the Hainan District of Wuhai City, Inner Mongolia, China (106°87′–106°89′E, 39°52′–39°55′N). This region falls within a temperate zone and experiences a continental climate characterized by cold winters, hot summers, minimal rainfall, strong winds, sandy terrain, and high thermal energy. The landscape is predominantly harsh and desert like.

### Soil sampling

In August 2019, three distinct desert plant communities in a protected area were selected as research sites. These included the community of *T. mongolica*, *Reaumuria songarica*, *Salsola passerine*, and *Stipa capillata* (Tm_Rs_Sp_S); *T. mongolica* and *S. capillata* (Tm_S); and the community dominated by *T. mongolica* (Tm) (**Supplementary Figure S1**). A large sample plot measuring 100 × 100 m was established for each plant community type, with approximately 1.2 km separating each plot. Within each large sample plot, five small plots (10 × 10 m) were arranged in a five-point pattern. All *T. mongolica* plants in the small plots were selected for soil collection. After removing 3 cm of topsoil, root zone soil samples (Rz_Soil) were collected at a radial distance of 5 cm from the plants at depths of 0–20 cm in four cardinal directions: southeast, southwest, northwest, and northeast. Simultaneously, a bare soil samples (B_soil) was collected from the area closest to *T. mongolica* that was devoid of roots. All the samples from the same small plot were combined into a single sample. Thirty soil samples were collected at each sampling site. All samples were stored on ice in sterile zip bags until arrival at the laboratory. Each sample was divided into two portions: one was stored at -80°C for molecular biology research, and the other was air-dried and sieved to determine its physical and chemical properties.

### Soil physicochemical properties

pH was measured using a calcium chloride extraction pH-sensitive electrode. Soil organic matter (SOM) content was measured using the potassium dichromate external heating method. Organic carbon (OC) was quantified using the potassium dichromate oxidation-external heating method. Total nitrogen (TN) content was determined via Kjeldahl nitrogen determination after the elimination of concentrated sulfuric acid and hydrogen peroxide. Organic nitrogen (ON) was measured using the hydrochloric acid hydrolysis-distillation method. Ammonium-nitrogen (NH_4_-N) was analyzed using the indigo blue colorimetric method after extracting the potassium chloride solution. Nitrate-nitrogen (NO_3_-N) was determined using the dual-wavelength colorimetric method after the extraction of the potassium chloride solution. The total phosphorus (TP) content was determined using the molybdenum-antimony resistance colorimetric method after digestion with concentrated sulfuric acid and hydrogen peroxide. The available phosphorus (AP) content was determined using the molybdate blue colorimetric method after extraction with a 0.5 M NaHCO_3_ solution. The total potassium (TK) content was measured using sodium hydroxide melt-flame spectrophotometry, and the available potassium (AK) was evaluated using ammonium acetate extraction-flame spectrophotometry. Water-soluble Ca^2+^, Mg^2+^, Na^+^, and K^+^ were quantified using deionized water extraction-flame spectrophotometry. Carbonate (CO_3_^2-^) was determined via deionized water extraction, followed by acid-alkali neutralization titration. Sulfate (SO_4_^2-^) was measured using deionized water extraction-barium sulfate turbidimetry, and chloride (Cl^-^) was determined through deionized water extraction-silver nitrate titration. These standard methods were followed, as described by Bao (2000) and Lu (2000).

### DNA extraction and library preparation

Total microbial genomic DNA was extracted from approximately 200 mg of soil using the E.Z.N.A.^®^ soil DNA Kit (Omega Bio-tek, Norcross, GA, US. PCR amplification of bacterial 16S rRNA and fungal ITS genes was conducted using Illumina-overhang-added primer pairs targeting the bacterial V4 region (515FmodF: 5’-GTGYCAGCMGCCGCGGTAA-3’ and 806RmodR: 5’-GGACTACNVGGGTWTCTAAT-3’) (Sampson et al., 2016; Walters et al., 2016) and fungal ITS1 region (ITS1F: 5’-CTTGGTCATTTAGAGGAAGTAA-3’ and ITS2R: 5’-GCTGCGTTCTTCATCGATGC-3’) (Sampson et al., 2016).

### Illumina MiSeq sequencing

Purified amplicons were pooled in equimolar amounts and paired-end sequenced on an Illumina MiSeq PE300 platform (Illumina, San Diego, CA, USA) following the standard protocols established by Majorbio Bio-Pharm Technology Co. Ltd. (Shanghai, China). The sequence reads were deposited in the NCBI Sequence Read Archive (SRA) database under accession numbers PRJNA1183314 and PRJNA1183552.

### Bioinformatics and statistical analysis

Bacterial and fungal OTUs were annotated using the Greengenes database (v13.8) (DeSantis et al., 2006) and the UNITE database (v8.2) (Nilsson et al., 2019), respectively, and reads that were not classified as bacterial or fungal were excluded. Bioinformatic analysis of soil bacteria and fungi was conducted using the Majorbio Cloud platform (https://cloud.majorbio.com). Microbial alpha diversity metrics, including Sobs, Shannon index, Simpson index, Heip evenness, Ace, and Chao1 richness, were estimated using Mothur v1.30.1 (Schloss et al., 2009). Both bacterial and fungal beta diversity analyses were performed using non-metric multidimensional scaling analysis (NMDS) with QIIME 2 (Bolyen et al., 2019) to calculate the distance matrix and the Vegan v2.5-3 package for analysis and plotting. Student’s *t*-test was used to evaluate differences in the relative abundance of microbial taxonomic groups. Venn diagram was created using the Venn diagram package in R (v1.6.20). Co-occurrence networks were constructed to explore internal community relationships across the samples (Barberán et al., 2012). A correlation between two nodes was considered statistically robust if Spearman’s correlation coefficient was >0.6 or <-0.6, with a *P*-value of <0.05. The functions of bacterial communities were predicted using the FAPROTAX tool (Louca et al., 2016). The functions of fungal communities were predicted using FUNGuild (http://www.funguild.org/). Student’s *t*-test was used to evaluate the differences in the functions of the microbial communities. Canonical correspondence analysis (CCA) and redundancy analysis (RDA) were performed using the Vegan v2.5-3 package to investigate the effects of soil physicochemical properties on the structure of the soil bacterial and fungal communities. The non-parametric permutational multivariate analysis of variance (PERMANOVA) test was used to assess the percentage of variation explained by the treatment, along with its statistical significance, using the vegan v2.5-3 package.

## Result

### Effect of *T. mongolica* on soil physicochemical properties

Various physicochemical characteristics of the samples were measured in the Rz_soil of *T. mongolica* in the three different plant communities (**Table 1**). In these communities, the soil pH in the *T. mongolica* Rz_soil decreased, whereas AK increased when compared with B_soil, which was sampled from areas without plant roots closest to *T. mongolica* plants. In both plant communities, SOM, OC, TN, nitrogen-to-phosphorus ratio (N/P), and K^+^ increased with the establishment of the *T. mongolica* plantations. Conversely, NH_4_-N and Na^+^ levels decreased in the *T. mongolica* plantations. NO_3_-N, Mg^2+^, and CO_3_^2-^ levels increased in *T. mongolica* plantations in only one plant community. ON, AP, and organophosphorus (OP) decreased in *T. mongolica* plantations in only one community. Across all three plant communities, the *T. mongolica* plantation had no significant effects on TP, TK, carbon-to-nitrogen ratio (C/N), carbon-to-phosphorus ratio (C/P), or Cl^-^. Ca^2+^ and SO_4_^2-^ levels increased in one community, decreased in the other, and showed no significant differences in the third community.

**Table 1:** Soil physicochemical properties in Rz_soil and B_soil in three plant communities in which *T. mongolica* is the dominant or keystone specie.

	Tm_Rs_Sp_S		Tm_S		Tm	
	Rz_soil	B_soil	Rz_soil	B_soil	Rz_soil	B_soil
pH	7.97 ± 0.19^b^	8.51 ± 0.17^a^	8.29 ± 0.14^b^	8.63 ± 0.11^a^	8.02 ± 0.08^b^	8.52 ± 0.12^a^
SOM (g/kg)	21.49 ± 4.03^a^	17.93 ± 0.81^b^	16.26 ± 3.79^a^	14.36 ± 1.48^a^	19.93 ± 2.28^a^	15.83 ± 3.24^b^
OC (g/kg)	12.47 ± 2.33^a^	10.40 ± 0.47^b^	9.43 ± 2.20^a^	8.33 ± 0.86^a^	11.56 ± 1.32^a^	9.18 ± 1.88^b^
TN (g/kg)	0.95 ± 0.07^a^	0.85 ± 0.43^a^	0.80 ± 0.11^a^	0.62 ± 0.08^b^	0.94 ± 0.16^a^	0.57 ± 0.16^b^
ON (mg/kg)	21.23 ± 10.01^b^	32.28 ± 10.75^a^	28.02 ± 16.16^a^	34.12 ± 10.14^a^	33.86 ± 10.91^a^	32.16 ± 8.23^a^
NO_3_-N (mg/kg)	4.69 ± 0.84^a^	4.89 ± 0.42^a^	5.70 ± 1.01^a^	4.84 ± 0.65^b^	5.35 ± 0.43^a^	5.03 ± 0.57^a^
NH_4_-N (mg/kg)	0.29 ± 0.09^a^	0.21 ± 0.11^a^	0.09 ± 0.03^b^	0.23 ± 0.03^a^	0.11 ± 0.01^b^	0.37 ± 0.15^a^
TP (g/kg)	0.70 ± 0.21^a^	0.61 ± 0.25^a^	0.44 ± 0.2^a^	0.55 ± 0.20^a^	0.52 ± 0.15^a^	0.60 ± 0.29^a^
AP (mg/kg)	1.39 ± 0.25^a^	1.44 ± 0.30^a^	1.50 ± 0.42^b^	2.14 ± 0.30^a^	1.67 ± 0.25^a^	1.85 ± 0.38^a^
OP (g/kg)	0.12 ± 0.06^a^	0.17 ± 0.08^a^	0.24 ± 0.04^a^	0.27 ± 0.02^a^	0.13 ± 0.04^b^	0.20 ± 0.04^a^
TK (g/kg)	3.39 ± 0.6^a^	3.98 ± 0.22^a^	2.93 ± 0.4^a^	3.25 ± 0.19^a^	3.02 ± 0.2^a^	3.22 ± 0.37^a^
AK (mg/kg)	245.72 ± 43.18^a^	191.74 ± 31.7^b^	182.53 ± 27.95^a^	148.23 ± 17.91^b^	214.70 ± 38.28^a^	168.66 ± 33.45^b^
N/P	1.47 ± 0.51^a^	1.58 ± 0.87^a^	2.31 ± 1.42^a^	1.34 ± 0.84^b^	1.92 ± 0.65^a^	1.08 ± 0.38^b^
C/N	13.18 ± 2.56^a^	15.04 ± 7.71^a^	12.09 ± 3.96^a^	13.75 ± 2.56^a^	12.64 ± 2.39^a^	17.2 ± 5.75^a^
C/P	19.45 ± 8.65^a^	19.78 ± 8.42^a^	24.69 ± 9.68^a^	17.46 ± 8.41^a^	23.43 ± 6.44^a^	18.53 ± 9.45^a^
Ca^2+^ (g/kg)	1.64 ± 0.16^a^	1.69 ± 0.11^a^	1.43 ± 0.07^b^	1.57 ± 0.07^a^	1.58 ± 0.10^a^	1.43 ± 0.08^b^
Mg^2+^ (g/kg)	0.55 ± 0.13^a^	0.56 ± 0.05^a^	0.73 ± 0.06^a^	0.50 ± 0.06^b^	0.59 ± 0.15^a^	0.61 ± 0.05^a^
Na^+^ (g/kg)	4.78 ± 0.11^b^	5.37 ± 0.29^a^	4.71 ± 0.15^b^	5.64 ± 1.05^a^	4.78 ± 0.19^a^	4.32 ± 1.05^a^
K^+^ (g/kg)	1.08 ± 0.15^a^	0.85 ± 0.14^b^	1.05 ± 0.25^a^	1.00 ± 0.23^a^	1.11 ± 0.21^a^	0.68 ± 0.2^b^
Cl^-^ (g/kg)	0.16 ± 0.03^a^	0.18 ± 0.07^a^	0.23 ± 0.06^a^	0.20 ± 0.06^a^	0.24 ± 0.07^a^	0.18 ± 0.06^a^
CO_3_^2-^ (g/kg)	0.29 ± 0.07^a^	0.26 ± 0.13^a^	0.35 ± 0.07^a^	0.23 ± 0.06^b^	0.25 ± 0.08^a^	0.28 ± 0.10^a^
SO_4_^2-^ (g/kg)	0.018 ± 0.001^a^	0.016 ± 0.001^b^	0.017 ± 0.001^a^	0.017 ± 0^a^	0.015 ± 0.002^b^	0.019 ± 0.002^a^

Different lowercase letters indicate significant differences between Rz_soil and B_soil based on Student’s t tests at p < 0.05. SOM, soil organic matter; OC, organic carbon; TN, total nitrogen; ON, organic nitrogen; NH_4_-N, ammonium-nitrogen; NO_3_-N, nitrate-nitrogen; TP, total phosphorus; AP, available phosphorus; OP, organic phosphorus; TK, total potassium; AK, available potassium; Ca^2+^, calcium ion; Mg^2+^, magnesium ion; Na^+^, sodium ion; K^+^, potassium ion; CO_3_^2-^, carbonate; SO_4_^2-^, sulfate; Cl^-^, chloride; C/N, carbon-to-nitrogen ratio; C/P, carbon-to-phosphorus ratio. Rz_soil, root zone soil; B_soil, bare soil; Tm_Rs_Sp_S, plant community of *T. mongolica*, *R. songarica*, *S. passerine*, and *S. capillata*; Tm_S, plant community of *T. mongolica* and *S. capillata*; Tm, plant community of *T. mongolica*.

### Effect of *T. mongolica* on α-diversity of bacterial and fungal communities in soil

The indices of α-diversity, including Sobs, Shannon, Chao, Pielou, and Coverage, were calculated to quantify the diversity, richness, evenness, and sequencing depth of the microbial communities in the three plant communities. The coverage index for all the microbial communities was approximately 0.9, indicating that the sequencing capability was acceptable (**Table 2**). *T. mongolica* significantly improved the Sobs and Ace diversity indices, as well as the Chao richness index, of bacterial and fungal communities in the Rz_soil compared with the B_soil. Shannon diversity and Heip evenness indices of the bacterial communities in the Rz_soil were significantly higher in the two plant communities, whereas the fungal communities showed significantly higher indices in only one plant community. However, there were no significant effects on the Simpson diversity index for either the bacterial or fungal communities across the three plant communities.

**Table 2:** α-diversity indices of bacterial and fungal communities in Rz_soil and B_soil in three plant communities in which *T. mongolica* is the dominant or keystone specie.

		Tm_Rs_Sp_S		Tm_S		Tm	
		Rz_soil	B_soil	Rz_soil	B_soil	Rz_soil	B_soil
Bacteria	Sobs	3220.60 ± 138.71^a^	2528.60 ± 252.85^b^	3376.20 ± 227.07^a^	2830.00 ± 64.83^b^	3585.00 ± 186.02^a^	2943.60 ± 154.48^b^
	Shannon	6.77 ± 0.07^a^	6.44 ± 0.14^b^	6.72 ± 0.17^a^	6.63 ± 0.03^a^	6.90 ± 0.08^a^	6.52 ± 0.17^b^
	Simpson	0.996 ± 0.001^a^	0.995 ± 0.001^a^	0.995 ± 0.001^a^	0.996 ± 0.001^a^	0.996 ± 0.001^a^	0.995 ± 0.002^a^
	Ace	4377.60 ± 184.7^a^	3401.30 ± 313.00^b^	4674.20 ± 293.54^a^	3952.50 ± 464.84^b^	4924.70 ± 250.14^a^	4088.70 ± 223.36^b^
	Chao	4345.10 ± 157.47^a^	3432.20 ± 333.68^b^	4659.40 ± 287.62^a^	3821.10 ± 202.69^b^	4897.00 ± 223.04^a^	4136.80 ± 288.96^b^
	Heip	0.27 ± 0.02^a^	0.25 ± 0.01^b^	0.25 ± 0.02^a^	0.27 ± 0.01^a^	0.28 ± 0.08^a^	0.23 ± 0.03^b^
	Coverage	0.96 ± 0^b^	0.97 ± 0^a^	0.96 ± 0^b^	0.97 ± 0^a^	0.96 ± 0^b^	0.97 ± 0^a^
Fungi	Sobs	366.80 ± 28.79^a^	228.80 ± 36.97^b^	366.80 ± 39.14^a^	194.60 ± 31.37^b^	457.80 ± 54.39^a^	198.80 ± 58.63^b^
	Shannon	3.83 ± 0.16^a^	3.54 ± 0.49^a^	3.64 ± 0.42^a^	3.47 ± 0.15^a^	3.73 ± 0.29^a^	3.32 ± 0.26^b^
	Simpson	0.95 ± 0.01^a^	0.93 ± 0.05^a^	0.93 ± 0.03^a^	0.93 ± 0.01^a^	0.93 ± 0.02^a^	0.92 ± 0.02^a^
	Ace	414.39 ± 25.37^a^	243.85 ± 38.55^b^	402.23 ± 51.02^a^	201.93 ± 35.95^b^	496.91 ± 62.71^a^	206.77 ± 57.04^b^
	Chao	418.25 ± 28.02^a^	253.77 ± 36.07^b^	409.68 ± 57.48^a^	203.49 ± 35.76^b^	496.62 ± 63.40^a^	207.45 ± 57.94^b^
	Heip	0.12 ± 0.02^a^	0.16 ± 0.05^a^	0.11 ± 0.05^a^	0.16 ± 0.01^a^	0.09 ± 0.02^b^	0.14 ± 0.04^a^
	Coverage	1.00 ± 0^a^	1.00 ± 0^a^	1.00 ± 0^a^	1.00 ± 0^a^	1.00 ± 0^a^	1.00 ± 0^a^

Different lowercase letters indicate significant differences between Rz_soil and B_soil based on Student’s t tests at p < 0.05. Rz_soil, root zone soil; B_soil, bare soil; Tm_Rs_Sp_S, plant community of *T. mongolica*, *R. songarica*, *S. passerine*, and *S. capillata*; Tm_S, plant community of *T. mongolica* and *S. capillata*; Tm, plant community of *T. mongolica*.

### Effect of *T. mongolica* on β-diversity of bacterial and fungal communities in soil

**Figure 1:**
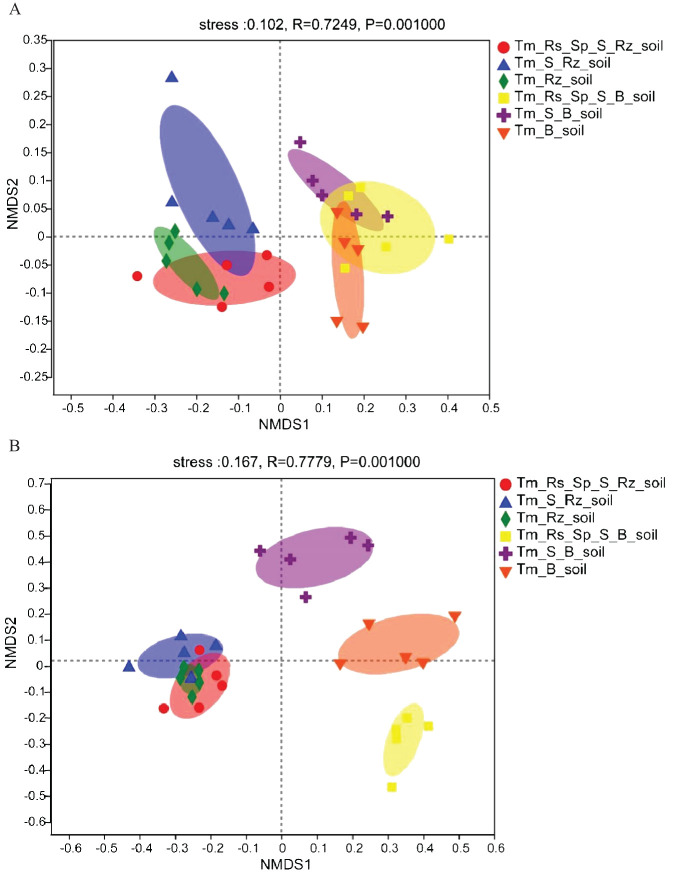
Non-metric multidimensional scaling (NMDS) analysis and plot of bacterial and fungal communities in Rz_soil and B_soil in three plant communities based on the bray–curtis distance. **(A)** The distribution of bacterial communities in Rz_soil and B_soil in three plant communities. **(B)** The distribution of fungal communities in Rz_soil and B_soil in three plant communities. Tm_Rs_Sp_S_Rz_soil, root zone soil in plant community of *T. mongolica*, *R. songarica*, *S. passerine*, and *S. capillata*; Tm_Rs_Sp_S_B_soil, bare soil in plant community of *T. mongolica*, *R. songarica*, *S. passerine*, and *S. capillata*; Tm_S_Rz_soil, root zone soil in plant community of *T. mongolica* and *S. capillata*; Tm_S_B_soil, bare soil in plant community of *T. mongolica* and *S. capillata*; Tm_Rz_soil, root zone soil in plant community of *T. mongolica*; Tm_B_soil, bare soil in plant community of *T. mongolica*.

All samples from Rz_soil and B_soil were positioned in distinct regions along the coordinate axis in the NMDS figure based on the Bray-Curtis method (stress <0.2). The bacterial and fungal communities in Rz_soil were separated from those in B_soil along the x-axis across the three plant communities (**Figure 1**). This finding demonstrates that *T. mongolica* plantations significantly altered the bacterial and fungal communities. However, the bacterial communities in the two soil types and the fungal communities in the Rz_soil were not distinctly different, and only the fungal communities in the B_soil exhibited a clear differentiation. This suggests that although the fungal communities in B_soil were distinct, the bacterial communities in B_soil showed less variation. In the *T. mongolica* plantation, both the bacterial and fungal communities in the Rz_soil exhibited minimal differences.

### Effect of *T. mongolica* on bacterial and fungal community composition in soil

*T. mongolica* did not significantly affect the number and diversity of bacterial and fungal phyla with a relative abundance of >1% in the B_soil and Rz_soil across the three plant communities. Actinobacteria was the dominant phylum in all samples. The relative abundances of Proteobacteria, Chloroflexi, Acidobacteria, Planctomycetes, Gemmatimonadetes, Verrucomicrobia, and Bacteroidetes were ranked from the second-most dominant phylum to the subsequent phyla (**Supplementary Figure S2A**). However, the relative abundance of these phyla varied between B_soil and Rz_soil in the three plant communities. Notably, the relative abundance of Bacteroidetes was significantly higher in the Rz_soil than in the B_soil across all three plant communities. Conversely, the relative abundance of Actinobacteria decreased, whereas the relative abundance of Proteobacteria increased significantly between Rz_soil and B_soil in Tm_Rs_Sp_S and Tm plant communities. These changes were not significant in the Tm_S community of the *T. mongolica* plantation. In addition, the relative abundance of Planctomycetes was greater in Rz_soil than in B_soil; however, this was only observed in the Tm_Rs_Sp_S plant community. In contrast, the relative abundance of Gemmatimonadetes was lower in Rz_soil than in B_soil, specifically in the Tm_S plant community (**Supplementary Figures S2B–D**).

Ascomycota was the dominant phylum in all samples. Basidiomycota, unclassified_k:fungi, and Mortierellomycota exhibited higher relative abundance (**Supplementary Figure S3A**). The relative abundance of phyla also varied between B_soil and Rz_soil across the three plant communities. Specifically, the relative abundance of Ascomycota increased, whereas the relative abundance of unclassified_k: fungi decreased significantly between Rz_soil and B_soil in the plant communities Tm_Rs_Sp_S and Tm. However, this change was not significant in the Tm_S plant community of the *T. mongolica* plantation. The relative abundances of Glomeromycota and Calcarisporiellomycota were lower in Rz_soil than in B_soil; however, this was only observed in the plant communities Tm_S and Tm (**Supplementary Figures S3B–D**).

**Figure 2:**
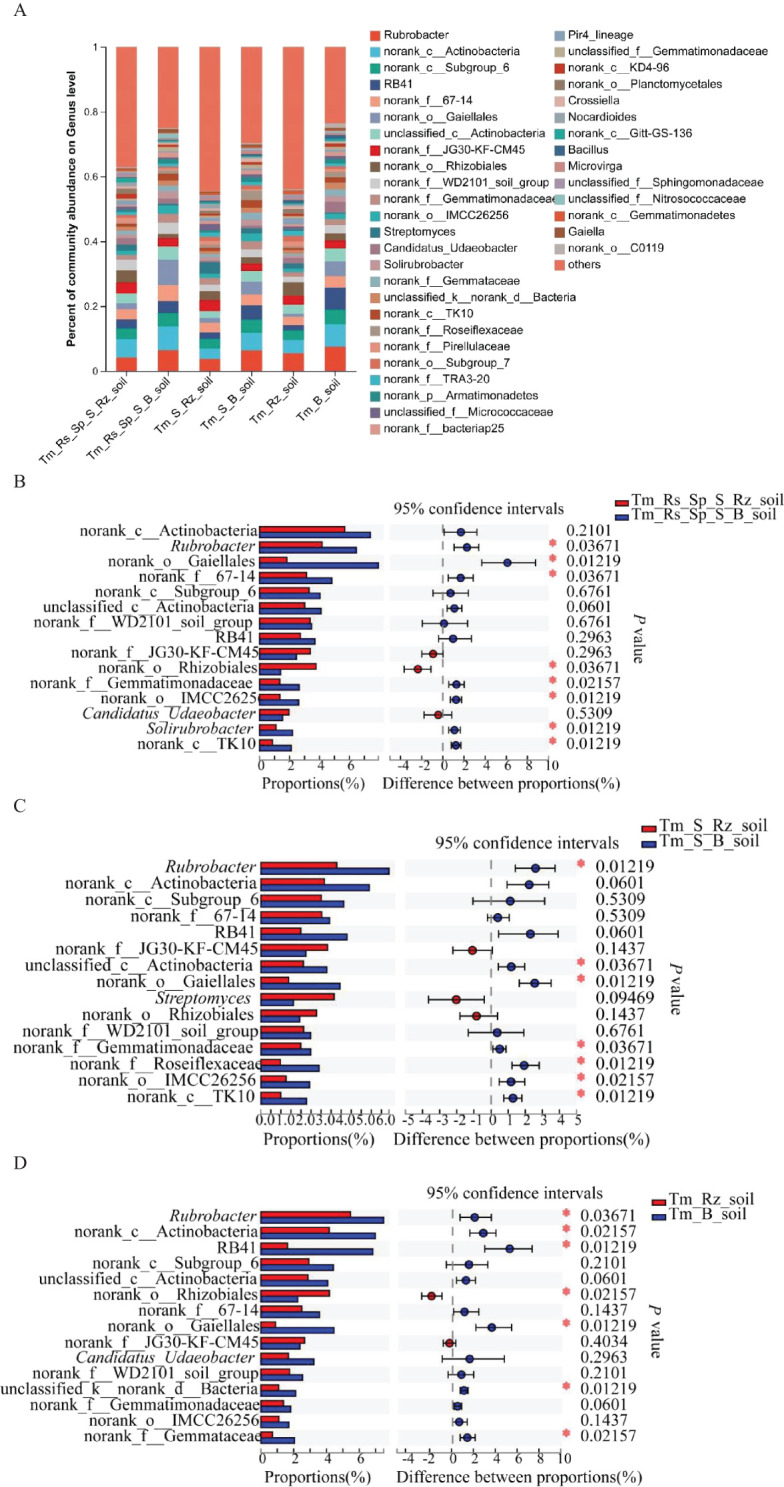
Relative abundance and differences of bacteria at genus level in Rz_soil and B_soil in three plant communities. **(A)** Genus-level bacterial communities’ composition. **(B–D)** Different bacterial genera in top 15 genera between B_soil and Rz_soil in the three plant communities. Tm_Rs_Sp_S_Rz_soil, root zone soil in plant community of *T. mongolica*, *R. songarica*, *S. passerine*, and *S. capillata*; Tm_Rs_Sp_S_B_soil, bare soil in plant community of *T. mongolica*, *R. songarica*, *S. passerine*, and *S. capillata*; Tm_S_Rz_soil, root zone soil in plant community of *T. mongolica* and *S. capillata*; Tm_S_B_soil, bare soil in plant community of *T. mongolica* and *S. capillata*; Tm_Rz_soil, root zone soil in plant community of *T. mongolica*; Tm_B_soil, bare soil in plant community of *T. mongolica*. * indicates a significant difference between Rz_soil and B_soil based on Student’s t tests at *p* < 0.05; ** indicates a significant difference at *p* < 0.01.

*T. mongolica* did not alter the dominant bacterial genera in the Rz_soil and B_soil across the three plant communities. *Rubrobacter* and norank_c:Actinobacteria were the dominant genera in all the soil samples from the three plant communities. However, the relative abundances of these two genera also changed between the B_soil and Rz_soil (**Figure 2A**). The relative abundances of *Rubrobacter* and norank_c:Actinobacteria declined significantly in the Rz_soil compared with those in the B_soil across the three plant communities (**Figures 2B–D**).

**Figure 3:**
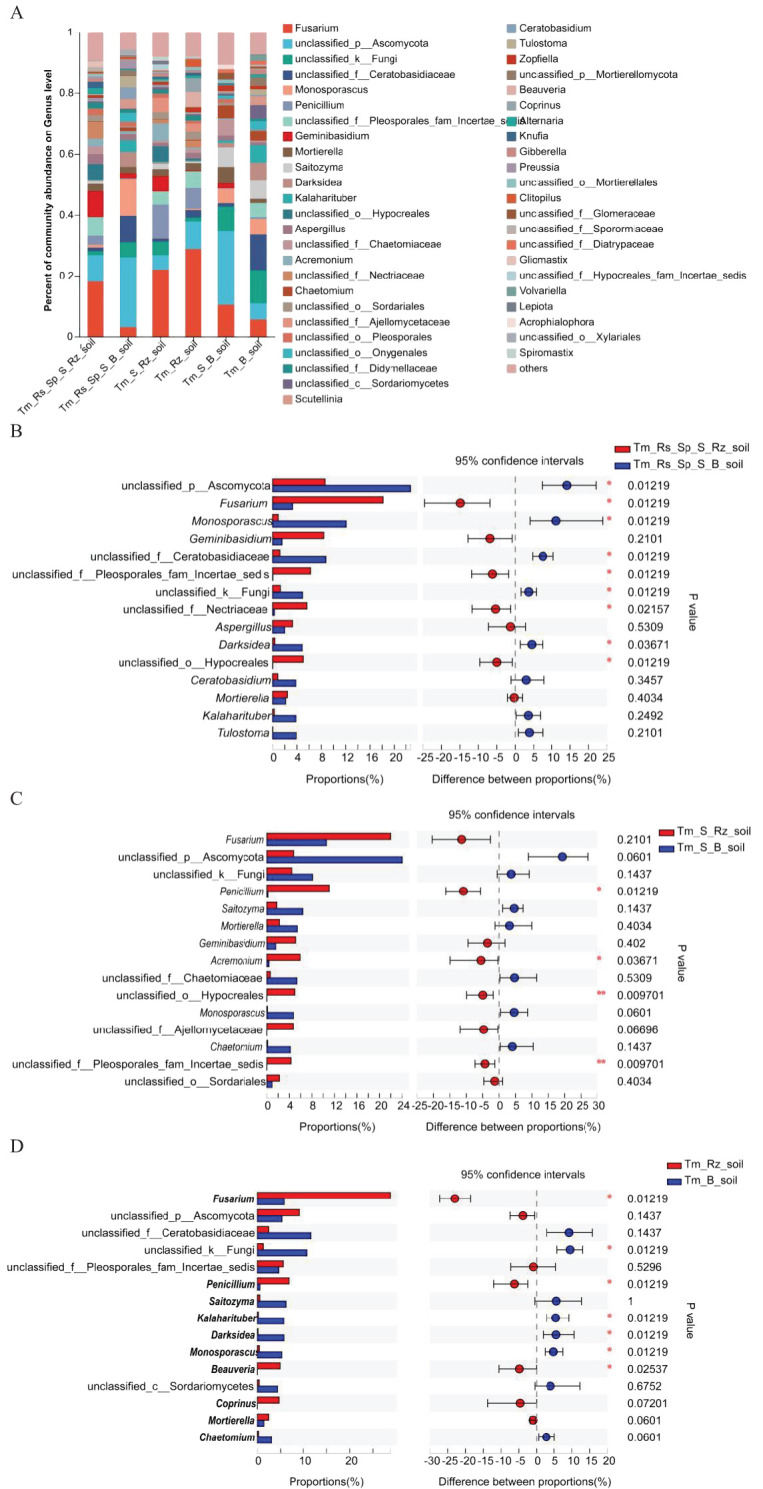
Relative abundance and differences of fungi at genus level in Rz_soil and B_soil in three plant communities. **(A)** Genus-level fungal communities’ composition. **(B–D)** Different fungal genera in top 15 genera between B_soil and Rz_soil from the three plant communities. Tm_Rs_Sp_S_Rz_soil, root zone soil in plant community of *T. mongolica*, *R. songarica*, *S. passerine*, and *S. capillata*; Tm_Rs_Sp_S_B_soil, bare soil in plant community of *T. mongolica*, *R. songarica*, *S. passerine*, and *S. capillata*; Tm_S_Rz_soil, root zone soil in plant community of *T. mongolica* and *S. capillata*; Tm_S_B_soil, bare soil in plant community of *T. mongolica* and *S. capillata*; Tm_Rz_soil, root zone soil in plant community of *T. mongolica*; Tm_B_soil, bare soil in plant community of *T. mongolica*. * indicates a significant difference between Rz_soil and B_soil based on Student’s t tests at *p* < 0.05; ** indicates a significant difference at *p* < 0.01.

*T. mongolica* altered the dominant fungal genera in the Rz_soil and B_soil across three plant communities. Unclassified p:Ascomycota was the dominant genus in B_soil in Tm_Rs_Sp_S and Tm_S plant communities. Unclassified f_Ceratobasidiaceae emerged as the dominant genus in the B_soil of the Tm plant community. *Fusarium* was the predominant genus in all Rz_soils of the three plant communities (**Figure 3A**). The relative abundances of *Fusarium*, unclassified f:Pleosporales_fam_Incertae_sedis, unclassified f:Nectriaceae, and unclassified o:Hypocreales were significantly higher in the Rz_soil than in the B_soil in the Tm_Rs_Sp_S plant community. Conversely, the relative abundances of unclassified p:Ascomycota, *Monosporascus*, unclassified f_Ceratobasidiaceae, unclassified k:Fungi, and *Darksidea* were lower in the Rz_soil than in the B_soil. In the Tm_S plant community, the relative abundances of *Penicillium*, *Acremonium*, unclassified f:Pleosporales_fam_Incertae_sedis, and unclassified o:Hypocreales were also higher in the Rz_soil than in the B_soil.

The relative abundances of *Fusarium, Penicillium*, and *Beauveria* were higher, whereas those of unclassified_k:Fungi, *Kalaharituber, Monosporascus*, and *Darksidea* were lower in Rz_soil than in B_soil from the Tm plant community (**Figures 3B–D**). At the phylum level, the relative abundance of Ascomycota increased, whereas that of unclassified_k _fungi decreased significantly in the Rz_soil and B_soil across the two plant communities. At the genus level, the relative abundances of *Fusarium* and *Penicillium* were higher, whereas those of *Monosporascus* and *Darksidea* were lower in the Rz_soil than in the B_soil in both plant communities.

In all three plant communities, the specific OTUs of bacteria and fungi in the Rz_soils were more abundant than those in the B_soils. Thus, the *T. mongolica* plantation enhanced the OTUs in the Rz_soil compared with the B_soil. In the bacterial communities, OTUs were more common than specific OTUs in both Rz_soil and B_soil. However, in the fungal communities, common OTUs were higher in the B_soil and lower in the Rz_soil (**Supplementary Figure S4**).

### Effect of *T. mongolica* on bacterial and fungal co-occurrence networks

**Figure 4:**
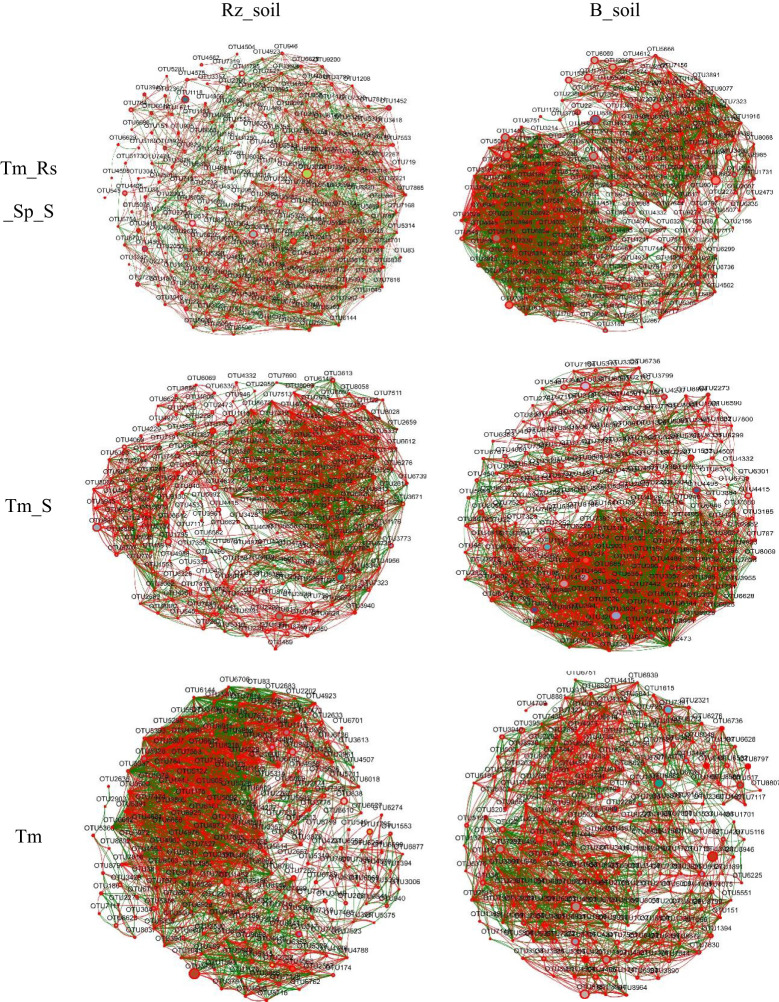
Bacterial co-occurrence networks between B_soil and Rz_soil in the three plant communities based on Spearman’s correlation coefficient (r) (|r| ≥ 0.5, *p <*0.05). The red line indicates positive correlation and the green line indicates negative correction. Rz_soil, root zone soil; B_soil, bare soil; Tm_Rs_Sp_S, plant community of *T. mongolica*, *R. songarica*, *S. passerine*, and *S. capillata*; Tm_S, plant community of *T. mongolica* and *S. capillata*; Tm, plant community of *T. mongolica*.

**Figure 5:**
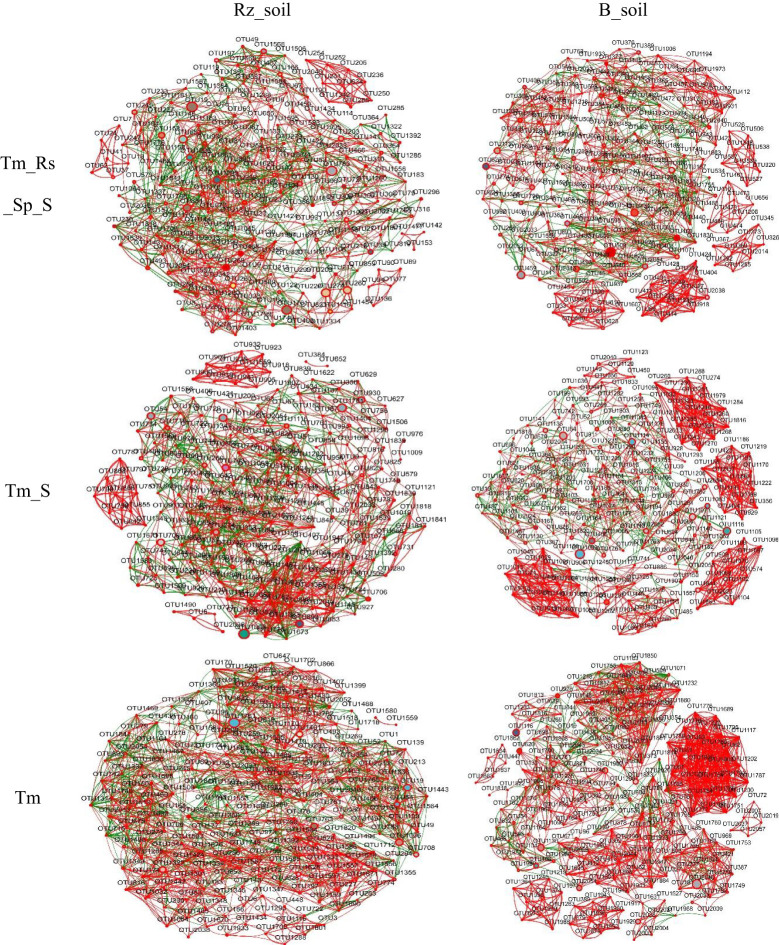
Fungal co-occurrence networks between B_soil and Rz_soil in the three plant communities based on Spearman’s correlation coefficient (r) (|r| ≥ 0.5, *p <*0.05). The red line indicates positive correlation and the green line indicates negative correction. Rz_soil, root zone soil; B_soil, bare soil; Tm_Rs_Sp_S, plant community of *T. mongolica*, *R. songarica*, *S. passerine*, and *S. capillata*; Tm_S, plant community of *T. mongolica* and *S. capillata*; Tm, plant community of *T. mongolica*.

Network analysis results indicated that the number of nodes, total edges, average degree, and density of the bacterial co-occurrence network in *T. mongolica* Rz_soil were lower than those in B_soil within Tm_Rs_Sp_S and Tm_S plant communities. However, in the Tm plant community, the co-occurrence network parameters in *T. mongolica* Rz_soil were higher than those in the B_soil. In contrast, for the fungal co-occurrence network, the parameters in *T. mongolica* Rz_soil exceeded those in B_soil across all three plant communities (**Figures 4**, **5**; **Supplementary Table S1**). For the bacterial co-occurrence network, the percentage of negative edges was similar to that of the positive edges. However, for the fungal co-occurrence network, the percentage of negative edges was considerably lower than that of positive edges. Therefore, *T. mongolica* appeared to decrease soil bacterial co-occurrence networks and enhance soil fungal co-occurrence networks.

### Effect of *T. mongolica* on bacterial and fungal community function

**Figure 6:**
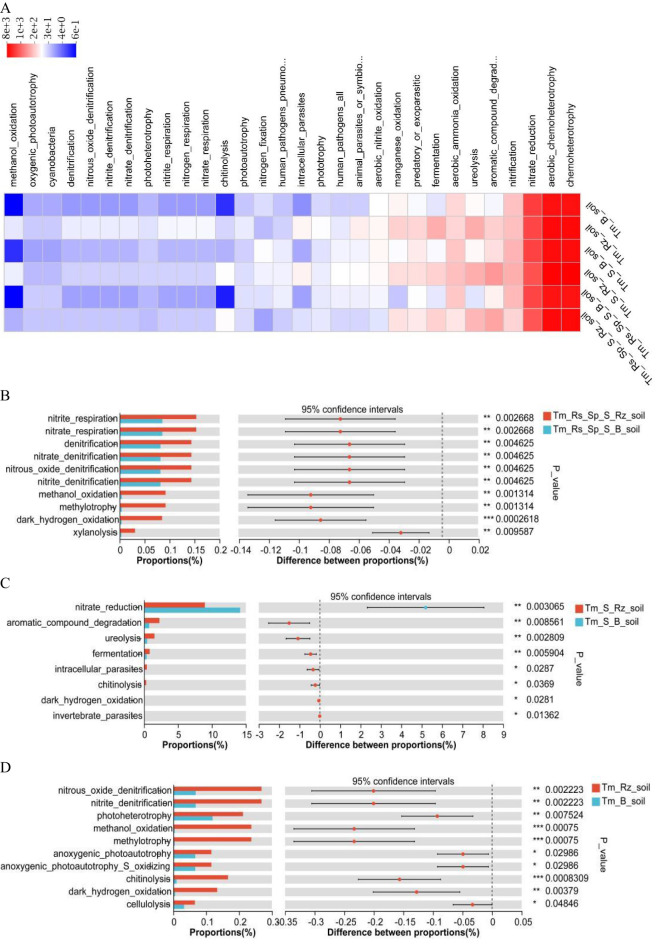
Bacterial community function predicted using the FAPROTAX tool in Rz_soil and B_soil in three plant communities. **(A)** Bacterial community function. **(B–D)** Bacterial community function in B_soil and Rz_soil in the three plant communities. Tm_Rs_Sp_S_Rz_soil, root zone soil in plant community of *T. mongolica*, *R. songarica*, *S. passerine*, and *S. capillata*; Tm_Rs_Sp_S_B_soil, bare soil in plant community of *T. mongolica*, *R. songarica*, *S. passerine*, and *S. capillata*; Tm_S_Rz_soil, root zone soil in plant community of *T. mongolica* and *S. capillata*; Tm_S_B_soil, bare soil in plant community of *T. mongolica* and *S. capillata*; Tm_Rz_soil, root zone soil in plant community of *T. mongolica*; Tm_B_soil, bare soil in plant community of *T. mongolica*. * indicates a significant difference between Rz_soil and B_soil based on Student’s t tests at *p* < 0.05; ** indicates a significant difference at *p* < 0.01; *** indicates a significant difference based on Student’s t tests at *p* < 0.001; **** indicates a significant difference based on Student’s t tests at *p* < 0.0001.

Functional roles of the bacterial community were predicted using the FAPROTAX tool (**Figure 6 A**). *T. mongolica* enhanced the functional capabilities of bacterial communities, including denitrification (nitrate respiration, nitrate denitrification, nitrite denitrification, and nitrous oxide denitrification), ureolysis, dark hydrogen oxidation, methylotrophy (methylotrophy and methanol oxidation), organic matter decomposition (xylanolysis, chitinolysis, and cellulolysis), aromatic compound degradation, and photoconversion (photoheterotrophy, anoxygenic photoautotrophy, and anoxygenic photoautotrophy S oxidizing) (**Figures 6B–D**). Consequently, *T. mongolica* facilitated soil functions related to energy acquisition through denitrification, methylotrophy, hydrogen oxidation, and photoconversion, while also being influenced by organic matter decomposition, aromatic compound degradation, and photoconversion.

**Figure 7:**
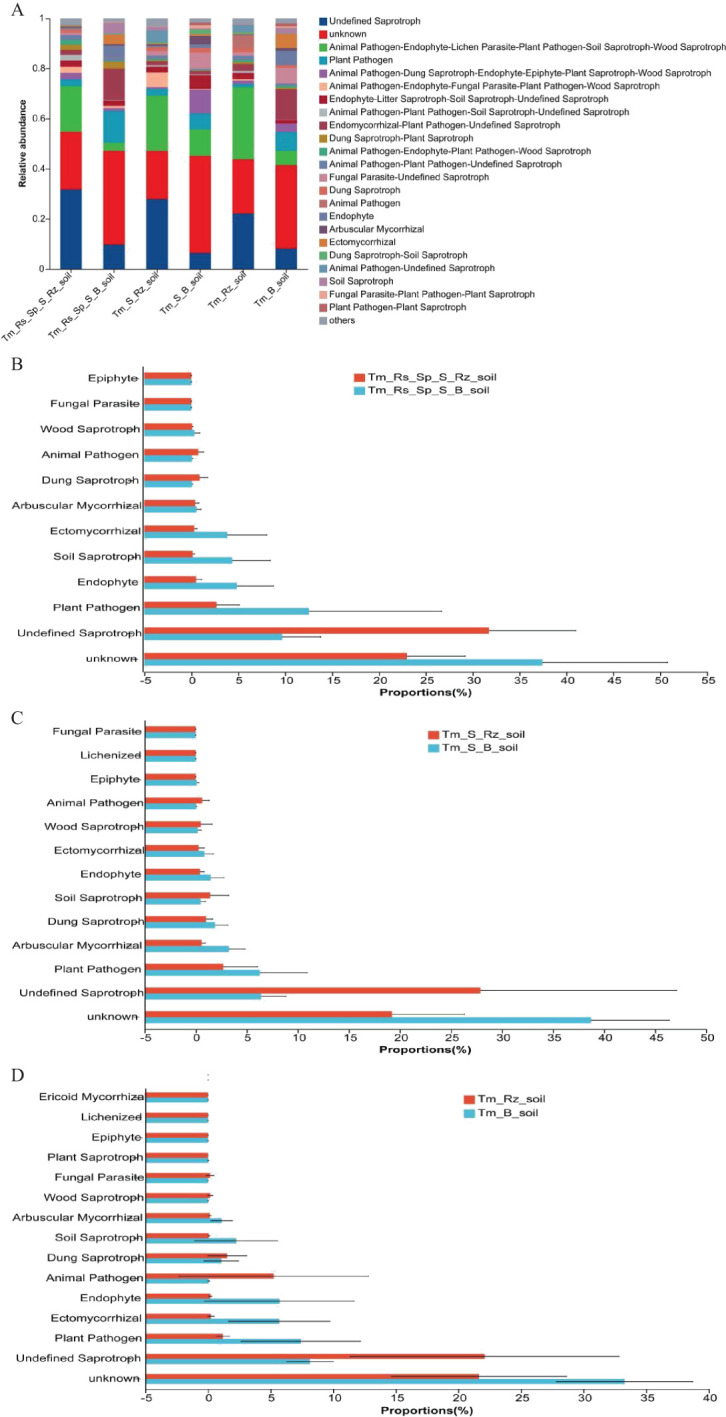
Fungal community function predicted using the FunGuild tool in Rz_soil and B_soil in three plant communities. **(A)** Fungal community function. **(B–D)** Fungal community function in B_soil and Rz_soil in the three plant communities. Error bars represent standard deviations (n = 5). Tm_Rs_Sp_S_Rz_soil, root zone soil in plant community of *T. mongolica*, *R. songarica*, *S. passerine*, and *S. capillata*; Tm_Rs_Sp_S_B_soil, bare soil in plant community of *T. mongolica*, *R. songarica*, *S. passerine*, and *S. capillata*; Tm_S_Rz_soil, root zone soil in plant community of *T. mongolica* and *S. capillata*; Tm_S_B_soil, bare soil in plant community of *T. mongolica* and *S. capillata*; Tm_Rz_soil, root zone soil in plant community of *T. mongolica*; Tm_B_soil, bare soil in plant community of *T. mongolica*.

The function of the fungal community was predicted using the FUNGuild tool. *T. mongolica* increased the relative abundance of undefined saprotrophs, animal pathogens, endophytes, lichen parasites, plant pathogens, soil saprotrophs, and wood saprotrophs whereas decreasing the relative abundance of plant pathogens within fungal communities. Thus, *T. mongolica* promoted the growth of saprotrophic fungi and reduced the prevalence of pathogenic fungi (**Figure 7A**). However, these changes were not significantly different between Rz_soil and B_soil across the three plant communities (**Figures 7B–D**).

### Factors driving bacterial and fungal communities in soil

**Figure 8:**
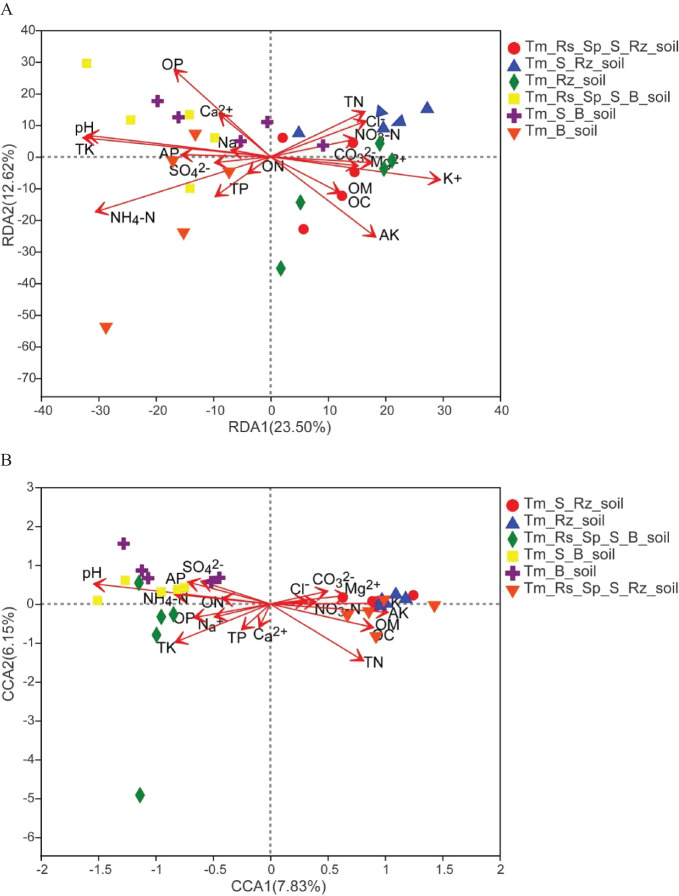
Canonical correspondence analysis (CCA) or redundancy analysis (RDA) of bacterial and fungal communities constrained by soil physicochemical properties. **(A)** RDA of the bacterial community with soil physicochemical properties. **(B)** CCA of the fungal community with soil physicochemical properties. SOM, soil organic matter; OC, organic carbon; TN, total nitrogen; ON, organic nitrogen; NH_4_-N, ammonium-nitrogen; NO_3_-N, nitrate-nitrogen; TP, total phosphorus; AP, available phosphorus; OP, organic phosphorus; TK, total potassium; AK, available potassium; Ca^2+^, calcium ion; Mg^2+^, magnesium ion; Na^+^, sodium ion; K^+^, potassium ion; CO_3_^2-^, carbonate; SO_4_^2-^, sulfate; Cl^-^, chloride. Tm_Rs_Sp_S_Rz_soil, root zone soil in plant community of *T. mongolica*, *R. songarica*, *S. passerine*, and *S. capillata*; Tm_Rs_Sp_S_B_soil, bare soil in plant community of *T. mongolica*, *R. songarica*, *S. passerine*, and *S. capillata*; Tm_S_Rz_soil, root zone soil in plant community of *T. mongolica* and *S. capillata*; Tm_S_B_soil, bare soil in plant community of *T. mongolica* and *S. capillata*; Tm_Rz_soil, root zone soil in plant community of *T. mongolica*; Tm_B_soil, bare soil in plant community of *T. mongolica*.

The effects of soil chemical properties on the bacterial and fungal communities were assessed using CCA and RDA. Among the 19 chemical properties examined, pH, TK, NH_4_-N, OP, AK, and K^+^ had a more significant effect on the bacterial community than the other properties. Specifically, pH, TK, NH_4_-N, and OP were positively correlated with B_soil whereas AK and K were negatively correlated with Rz_soil. OM, pH, TK, TN, AK, AP, OC, and K^+^ significantly affected the fungal community. pH was the most influential factor, showing a positive correlation with B_soil and a negative correlation with Rz_soil (**Figure 8**).

## Discussion

### *T. mongolica* has a significant influence on soil

Plants have a significant influence on the soil. This influence is multifaceted, encompassing the enhancement of the physical, chemical, and biological properties of the soil as well as the cycling and utilization of nutrients and soil microbes. Collectively, these factors promote the stability and sustainable development of ecosystems (Kranz et al., 2020; Liu et al., 2020; Furey and Tilman, 2021). This study demonstrated that *T. mongolica* reduced soil pH by decreasing Na^+^ and K^+^ content, increased AK, SOM, OC, and TN in desert soil. As a keystone species in desert communities, *T. mongolica* plays an irreplaceable role in maintaining local ecosystem stability and protecting the ecological environment through “plant-soil feedback.”

### Soil physicochemical properties changed by *T. mongolica*

During plant growth, both acidic and alkaline substances are released, which can influence the acidity or alkalinity of soil. For instance, certain plants release organic acids during their growth process, resulting in increased soil acidity, whereas other plants may enhance soil alkalinity through root exudates (Tibbett et al., 2019). Yan et al. (2020) observed that tea plantations contribute to soil acidification, with pH levels decreasing by 0.47 to 1.43. In the fruit and vegetable systems, the pH decreased by 0.40 to 1.08, and in the cereal systems, it decreased by 0.30 to 0.89. Zhou et al. (2017) reported the pH was 4.58 in 78-year-old forest plantations of slash pine, 5.74 in Hoop pine, 6.01 in Kauri pine, and 4.49 in Eucalyptus. Jin et al. (2022) demonstrated that hickory plantations increased the pH of relatively acidic soils, but decreased the pH of relatively alkaline soils. The *T. mongolica* plantation also reduced the pH by 0.5 across the three plant communities. The environment in which *T. mongolica* thrives is the irrigation area in the middle and upper reaches of the Yellow River, characterized by long-term dryness, minimal rainfall, and high evaporation rates. Soil salinity is primarily composed of sulfates and chlorides. An arid climate and improper irrigation practices have led to the secondary salinization of the soil. The concentrations of water-soluble neutral salts, such as NaCl and Na_2_SO_4_, in the soil exceed 0.1%, whereas the concentrations of alkaline salts, such as Na_2_CO_3_ and NaHCO_3_, exceed 5% (Li et al., 2023). Consequently, the soil is classified as severely saline-alkali. Our findings indicate that *T. mongolica* significantly decreased soil salinity (**Table 1**). Na^+^ concentration decreased significantly in the *T. mongolica* plantation across both plant communities. However, the concentrations of Cl^-^, SO_4_^2-^, and CO_3_^2-^ were not reduced. Therefore, *T. mongolica* lowered soil pH by decreasing Na^+^ concentrations.

Plants can loosen soil, increase soil porosity, reduce soil compaction, and enhance soil physical properties via root activity. Plant residues and root exudates can increase the organic matter content of soil (Oleghe et al., 2019). Changes in soil acidity or alkalinity can influence the solubility and availability of mineral elements, thereby affecting their absorption and utilization by plants (Zhao et al., 2021). Our findings indicate that *T. mongolica* increased SOM and OC in the Rz_soil of *T. mongolica* compared with B_soil. We hypothesized that *T. mongolica* enhances C content in the soil through root sediments, root secretions, and litter. TN also increased; however, ON and NH_4_-N levels decreased. We speculate that *T. mongolica* contributes to the N content in the soil through nitrogen fixation and recovery. In addition, *T. mongolica* appeared to accelerate the decomposition of ON and absorption of NH_4_-N; however, it did not affect TP or AP transformation. In only one plant community was P absorption enhanced, leading to reduced AP levels. Although, *T. mongolica* did not affect TK, it increased AK by accelerating K release.

### Soil bacterial and fungal abundance and community due to *T. mongolica*

The organic matter produced by the *T. mongolica* plantation not only provides abundant carbon and energy sources for soil microorganisms but also enhances soil structure and increases soil biodiversity. In this study, we found that the bacterial and fungal communities in Rz_soil were distinct from those in B_soil across the three plant communities within the *T. mongolica* plantation. *T. mongolica* significantly improved the diversity index (Sobs) and the richness index (Chao) of both bacterial and fungal communities in the three plant communities. In this study, *Rubrobacter* and norank_c_Actinobacteria were the dominant genera across all the soil samples collected from the three plant communities. The presence of *T. mongolica* did not alter the dominant bacterial genera in the Rz_soil or B_soil of these communities. However, *T. mongolica* plantations affected the relative abundances of certain genera. Specifically, the relative abundance of *Rubrobacter* and norank_c_Actinobacteria significantly declined in the Rz_soil compared with that in the B_soil across the three plant communities. Both *Rubrobacter* and norank_c_Actinobacteria belong to the phylum Actinobacteria, which is known for its potential to resist plant diseases, promote plant growth, and enhance salt tolerance in plants (Passari et al., 2019; Xiong et al., 2019). The small perennial shrub *T. mongolica* decreases the abundance of beneficial bacteria in long-term plantations, a phenomenon commonly observed in crop cultivation (Schlatter et al., 2017; Lee et al., 2021).

In this study, we found that the unclassified phylum Ascomycota was the dominant genus in B_soils in Tm_Rs_Sp_S and Tm_S. The unclassified family Ceratobasidiaceae emerged as the dominant genus in the B_soil from the Tm plant community. *Fusarium* was the predominant genus in all Rz soils across all three plant communities. *Fusarium* is a potential fungal pathogen (van Agtmaal et al., 2017). The increased abundance of *Fusarium*, a potential plant fungal pathogen, requires the recruitment of antagonistic bacteria to maintain plant health. Additionally, *T. mongolica* altered the relative abundance of the dominant bacterial genera *Rubrobacter* and norank_c_Actinobacteria in both Rz_soils and B_soils in the three plant communities. The relative abundances of *Rubrobacter* and norank_c_Actinobacteria were significantly lower in Rz_soil than in B_soil across the three plant communities. Consequently, *T. mongolica* poses a high risk for root disease outbreaks.

The high-throughput sequencing technology employed in this study is capable of delineating the species and relative abundance of microbial community structure. However, it is crucial to acknowledge that relative abundance alone may not provide a comprehensive reflection of microbial quantities, particularly when assessing microbial community. To address this limitation, absolute abundance can be suggested. Jiang et al. (2019) observed that the relative abundance of Proteobacteria remained statistically unchanged in fertilizer application experiments conducted on tomato crops in coastal saline-alkali soil. Nonetheless, when absolute abundance quantification methods can be utilized, a significant escalation in the absolute abundance of Proteobacteria was detected. Similar findings have been documented in the studies by Yang et al. (2018) and Lou et al. (2018). Consequently, absolute quantitation of microbiota abundance is essential.

Methods of absolute quantitation of microbiota abundance encompass a variety of techniques, including the use of spike-in bacteria as documented by Stammler et al. (2016), the quantification of bacterial DNA through Flow Cytometry (FCM) as outlined by Vandeputte et al. (2017), and the integrated high-throughput absolute abundance quantification (iHAAQ) approach, which merges high-throughput sequencing with quantitative Polymerase Chain Reaction (qPCR), as introduced Lou et al. (2018). Additionally, Tkacz et al. (2018) presented an absolute quantitation method utilizing synthetic spike DNA. Guo et al. (2020) employed host-associated quantitative abundance profiling (HA-QAP) to delineate variations in the microbial load within the root microbiome. Additionally, Zhang et al. (2022) assessed two absolute microbiome profiling (AMP) methods in soil microbiota quantitative research, spike-AMP and qPCR-AMP, concluding that qPCR-AMP is superior for quantitative assessment of soil microbial communities. Wang et al. (2020) proposed an “amplification-selection” model for rhizosphere microbiome assembly, which utilizes synthetic chimeric spikes in plasmids for microbiome profiling. They hypothesized that plant root exudates can ‘feed’ soil microorganisms, nutrition-poor bulk soil is seen as “countryside”, while rhizosphere soil is as “metropolis”, where microbial communities can find more opportunities for growth and reproduction. The microorganisms in rhizosphere soil undergo amplification prior to the selection by the root system.

In this study, we observed *T. mongolica* notably enhanced the diversity indices (Sobs and Ace), as well as the richness index (Chao), of both bacterial and fungal communities across three plant communities. Moreover, the relative abundance of Bacteroidetes and Ascomycota in the Rz_soils was significantly higher than that in the B_soils. These findings aligned with “two-step or multiple-step selection” model, as inferred from the relative abundance data obtained through amplicon-based high-throughput sequencing. Therefore, absolute quantitation of microbiota abundance should be recommended in microbial ecology.

### Soil properties driving soil bacterial and fungal communities

In this study, pH, TK, NH_4_-N, OP, AK, and K had a more significant effect on the bacterial community in the soil than the other chemical properties. The pH, TK, NH_4_-N, and OP were positively correlated with B_soil, whereas AK and K were negatively correlated with Rz_soil. For the fungal community, pH had the most substantial effect, showing a positive correlation with B_soil and a negative correlation with Rz_soil. Zhou et al. (2017) asserted that pH and vegetation are the primary factors influencing soil bacterial diversity and composition in the chronosequence of rubber trees (*Hevea brasiliensis*) plantations. Lauber et al. (2009) reported that soil pH significantly affects the structure of soil bacterial communities on a continental scale. Different microorganisms thrive within specific pH ranges, which are conducive to their growth and reproduction. Consequently, soil pH can influence the activity of soil microorganisms; if the pH of the soil solution falls outside the appropriate range, microbial activity is inhibited. Furthermore, the pH of the soil solution can alter the solubility of minerals, thereby affecting the nutrient availability in the soil and the activity of soil microorganisms (Philippot et al., 2024).

### Mechanism of *T. mongolica* influence on soil

Soil enzymes play a crucial role in soil organic matter degradation, mineralization, and nutrient cycling. Their activities significantly influence soil nutrient content (Burns et al., 2013; Dotaniya et al., 2019). By measuring the soil enzyme activity, we can gain a deeper understanding of how *T. mongolica* affects soil properties from a protein perspective. Unfortunately, the soil enzyme activity between Rz_soil and B_soil in soil samples from three plant communities. It is one of further research work.

Microorganism in soil is another biological factor that affects soil nutrition (Zhang et al., 2021; Coban et al., 2022; Philippot et al., 2024). The high-throughput sequencing technology is common method for reveal microorganism in soil (Nkongolo and Narendrula-Kotha, 2020). In this study, throughput sequencing technology based on amplicon was used to determine microbial communities. However, metagenomic sequencing technology, which is superior to amplicon sequencing, can provide insights into bacteria and fungi involved in biogeochemical cycles, not only soil microbial community composition, but also their functional genes and metabolic pathways. This is particularly for the C cycle (including CO_2_ fixation and respiration), N cycle (encompassing nitrification, denitrification, and N_2_ fixation), P cycle, and S cycle (including sulfur assimilation, anaerobic sulfate respiration, and sulfide oxidation) (Simon and Daniel, 2011; Scholz et al., 2012). Further research in this area is required.

## Conclusion

*T. mongolica* is rooted in the West Ordos Region of Inner Mongolia, northwest China, since the ancient Mediterranean period, approximately 140 million years ago. This study found that *T. mongolica* plantations decreased the soil pH and increased the nutrient content. Additionally, *T. mongolica* plantations altered community composition, co-occurrence networks, and ecological functions. In conclusion, as a keystone species in desert ecosystems, *T. mongolica* plantations significantly influence desert soil properties and microbial communities and play an irreplaceable role in local ecosystem stability. These findings offer a new perspective to understand the role of *T. Mongolica* in the desert ecosystems.

## Data Availability

The datasets presented in this study can be found in online repositories. The names of the repository/repositories and accession number(s) can be found in the article/**Supplementary Material**.
