# Supplementary material for: The relict plant Tetraena mongolica plantations increase the nutrition and microbial diversity in desert soil
Source: Front Plant Sci. 2025 Mar 20;16:1539336. doi: 10.3389/fpls.2025.1539336 (PMC11965594; doi:10.3389/fpls.2025.1539336)
Supplement: Supplementary file 1 [file DataSheet1.docx]

**The relict plant** ***Tetraena mongolica* plantations increase the nutrition and microbial diversity in desert soil**

Yanan Quan^1,2,3^, Xiuwen Gan^1,2,3†^, Shiyun Lu^1,2,3^, Xiaodong Shi^1,2,3^, Mingsheng Bai^1,2,3^, Ying Lin^1,2,3^, Yufei Gou^1,2,3^, Hong Zhang^1,2,3^, Xinyue Zhang^1,2,3^, Jiayuan Wei^1,2,3^, Tianyu Chang^1,2,3^, Jingyu Li^1,2,3^, Jianli Liu^1,2,3^*

1.College of Biological Science and Engineering, North Minzu University, Yinchuan, Ningxia, China

2.Key Laboratory of Ecological Protection of Agro-pastoral Ecotones in the Yellow River Basin, National Ethnic Affairs Commission of the People’s Republic of China, Yinchuan, Ningxia, China

3.Ningxia Key Laboratory of Microbial Resources Development and Applications in Special Environment, Science and Technology Department of Ningxia, Yinchuan, Ningxia, China

*****Corresponding author: Jianli Liu

E-mail: ljl7523@126.com

**Number of figures: 4**

**Supplementary Material -Figures**

| A  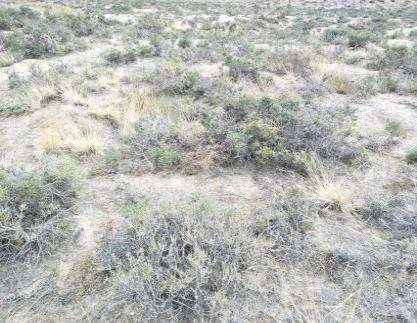 | B  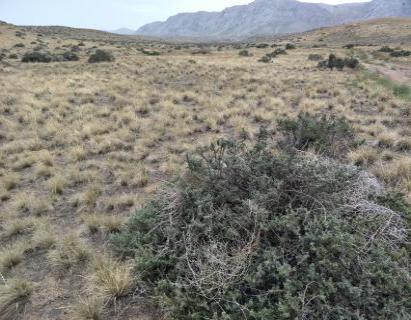 | C  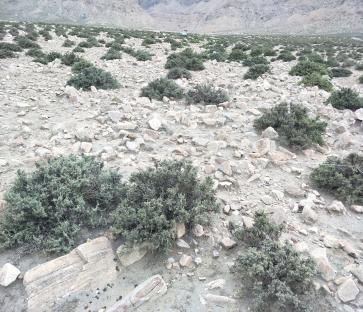 |
| --- | --- | --- |

**Fig. S1.** Schematic of experimental plots. **(A)** Plant community of *T. mongolica*, *R. songarica*, *S. passerine,* and *S. capillata* (Tm_Rs_Sp_S); **(B)** Plant community of *T. mongolica* and *S. capillata* (Tm_S); **(C)** Plant community of *T. mongolica* (Tm).

| A  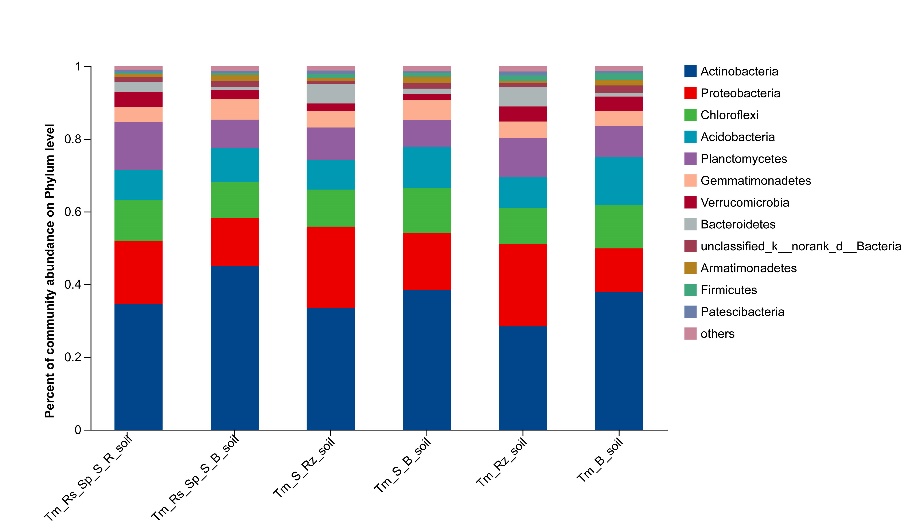 |
| --- |
| B  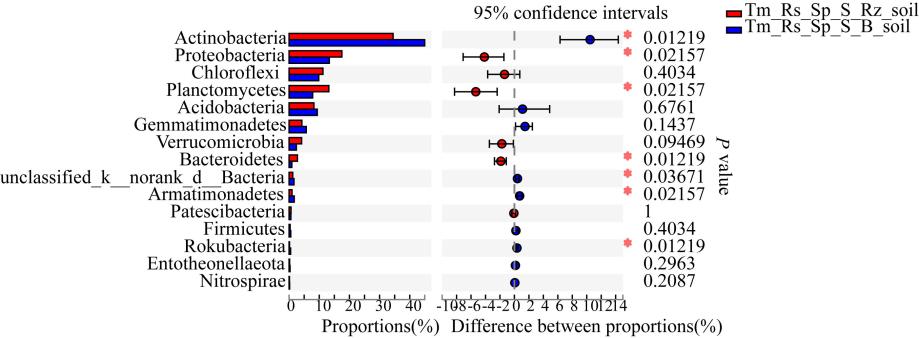 |
| C  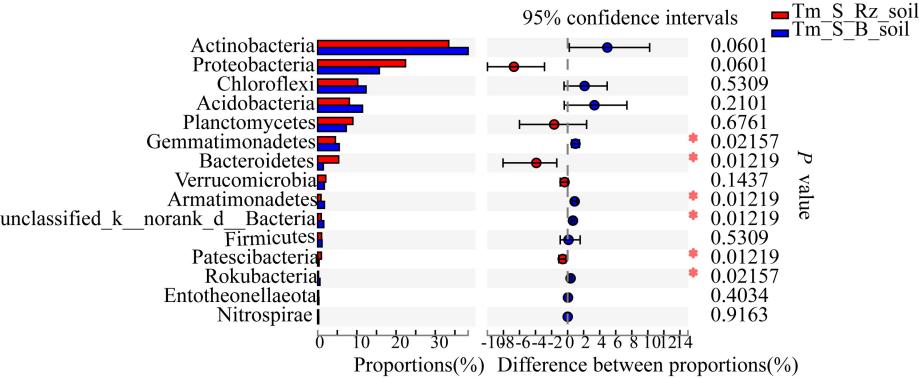 |
| D  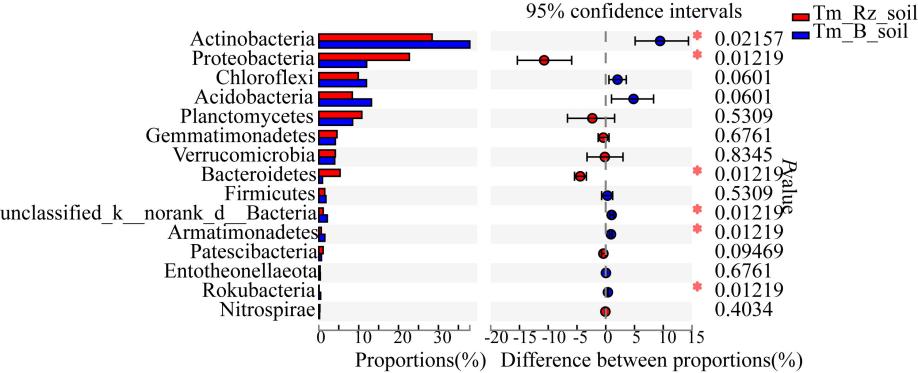 |

**Fig. S2.** Relative abundance and differences of bacteria at phylum level in Rz_soil and B_soil in three plant communities. **(A)** Phylum-level bacterial communities’ composition. **(B- D)** Different bacterial phyla between B_soil and Rz_soil in the three plant communities. Tm_Rs_Sp_S_Rz_soil, root zone soil in plant community of *T. mongolica*, *R. songarica*, *S. passerine,* and *S. capillata*; Tm_Rs_Sp_S_B_soil, bare soil in plant community of *T. mongolica*, *R. songarica*, *S. passerine,* and *S. capillata*; Tm_S_Rz_soil, root zone soil in plant community of *T. mongolica* and *S. capillata*; Tm_S_B_soil, bare soil in plant community of *T. mongolica* and *S. capillata*; Tm_Rz_soil, root zone soil in plant community of *T. mongolica*; Tm_B_soil, bare soil in plant community of *T. mongolica*. * indicates a significant difference between Rz_soil and B_soil based on Student's t tests at *p* < 0.05.

| A  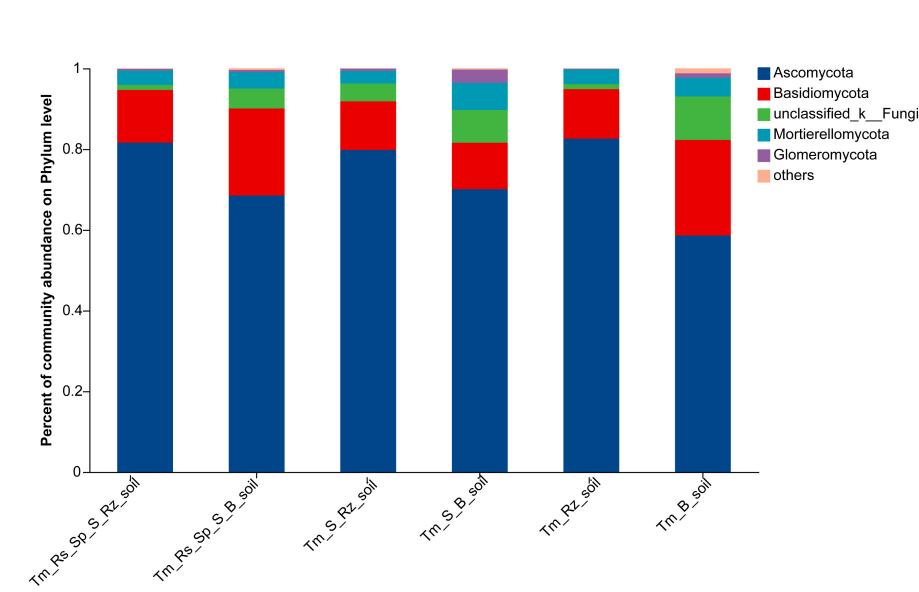 |
| --- |
| B  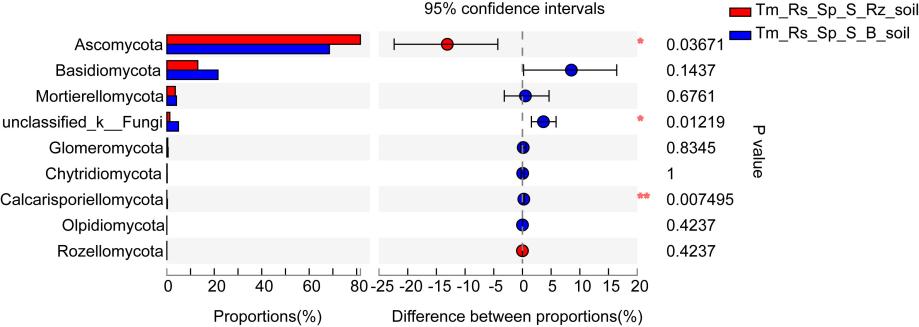 |
| C  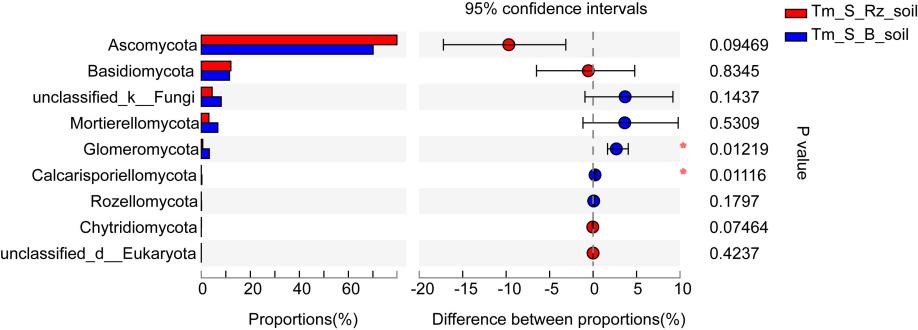 |
| D  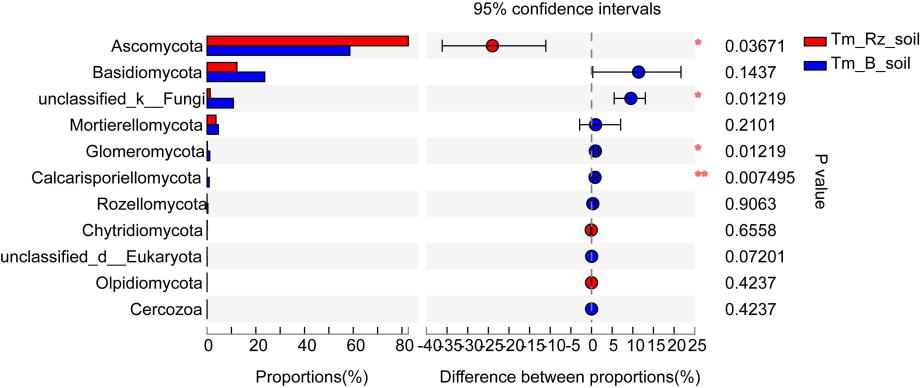 |

**Fig. S3.** Relative abundance and differences of fungal at phylum level in Rz_soil and B_soil in three plant communities. **(A)** Phylum-level fungal communities’ composition. **(B- D)** Different bacterial phyla between B_soil and Rz_soil in the three plant communities. Tm_Rs_Sp_S_Rz_soil, root zone soil in plant community of *T. mongolica*, *R. songarica*, *S. passerine,* and *S. capillata*; Tm_Rs_Sp_S_B_soil, bare soil in plant community of *T. mongolica*, *R. songarica*, *S. passerine,* and *S. capillata*; Tm_S_Rz_soil, root zone soil in plant community of *T. mongolica* and *S. capillata*; Tm_S_B_soil, bare soil in plant community of *T. mongolica* and *S. capillata*; Tm_Rz_soil, root zone soil in plant community of *T. mongolica*; Tm_B_soil, bare soil in plant community of *T. mongolica*. * indicates a significant difference between Rz_soil and B_soil based on Student's t tests at *p* < 0.05; ** indicates a significant difference at *p* < 0.01.

| A  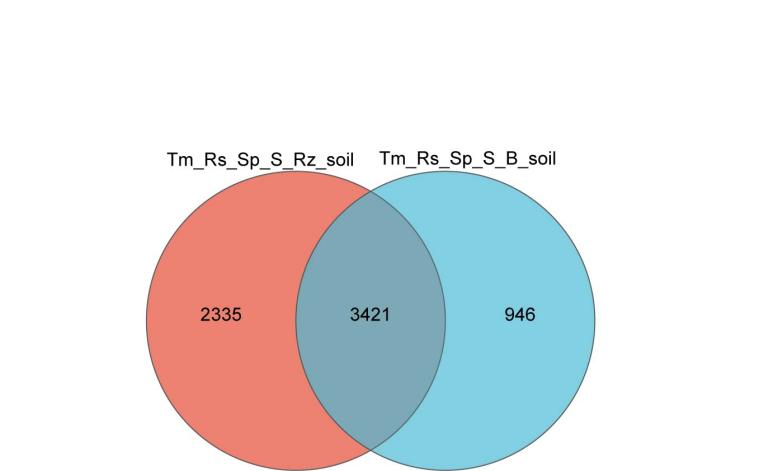 | B  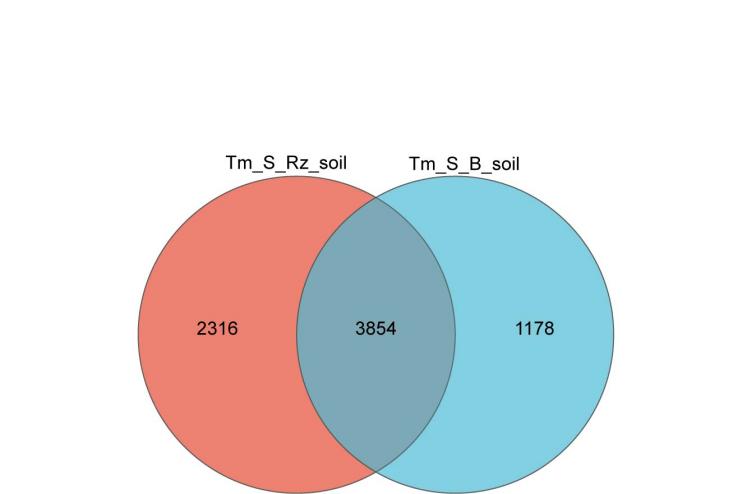 | C  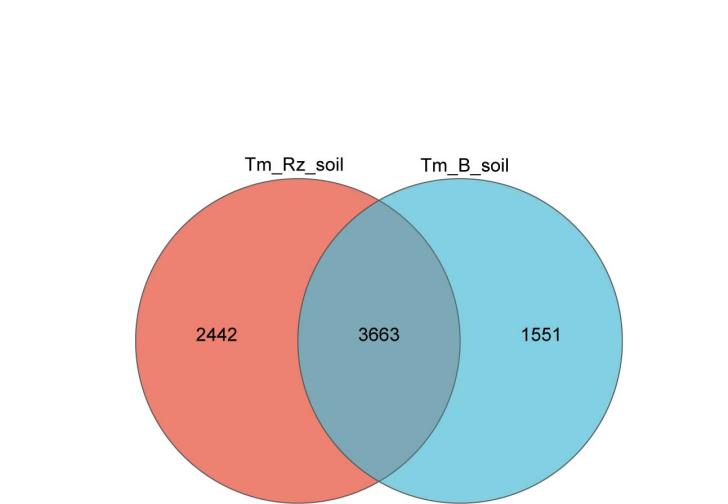 |
| --- | --- | --- |
| D  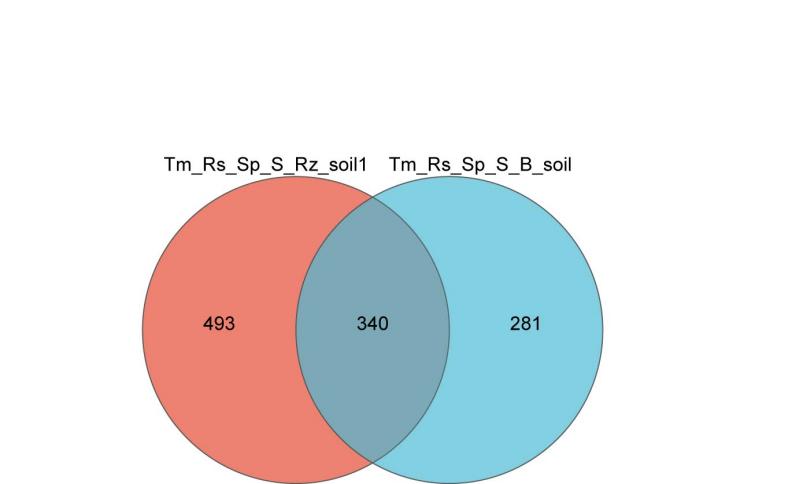 | E  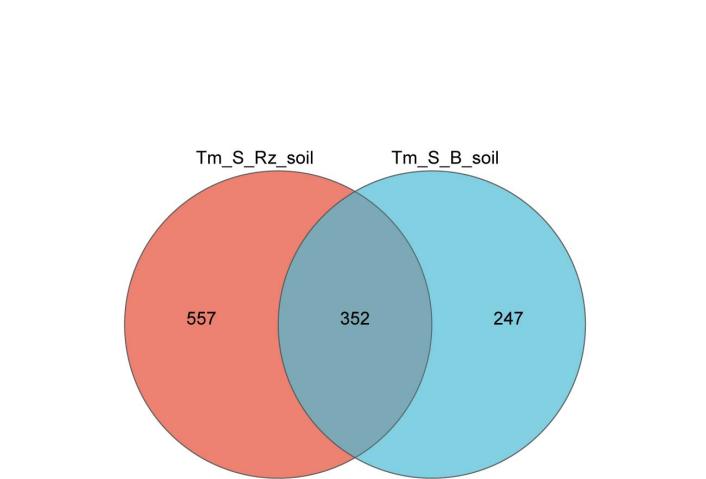 | F  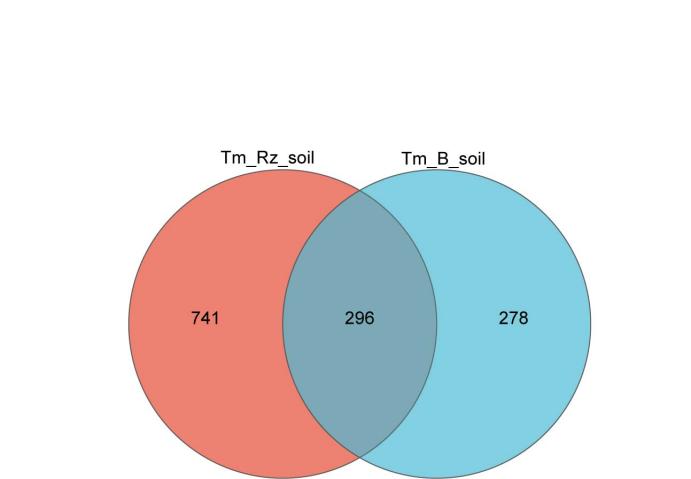 |

Fig. S4. Venn of bacterial and fungal communities between B_soil and Rz_soil in the three plant communities. **(A-C)** Bacterial communities. **(D-F)** Fungal community. Tm_Rs_Sp_S_Rz_soil, root zone soil in plant community of *T. mongolica*, *R. songarica*, *S. passerine,* and *S. capillata*; Tm_Rs_Sp_S_B_soil, bare soil in plant community of *T. mongolica*, *R. songarica*, *S. passerine,* and *S. capillata*; Tm_S_Rz_soil, root zone soil in plant community of *T. mongolica* and *S. capillata*; Tm_S_B_soil, bare soil in plant community of *T. mongolica* and *S. capillata*; Tm_Rz_soil, root zone soil in plant community of *T. mongolica*; Tm_B_soil, bare soil in plant community of *T. mongolica*.
