# Supplementary material for: The relict plant Tetraena mongolica plantations increase the nutrition and microbial diversity in desert soil
Source: Front Plant Sci. 2025 Mar 20;16:1539336. doi: 10.3389/fpls.2025.1539336 (PMC11965594; doi:10.3389/fpls.2025.1539336)
Supplement: Supplementary file 2 [file DataSheet2.docx]

**The relict plant** ***Tetraena mongolica* plantations increase the nutrition and microbial diversity in desert soil**

Yanan Quan^1,2,3^, Xiuwen Gan^1,2,3†^, Shiyun Lu^1,2,3^, Xiaodong Shi^1,2,3^, Mingsheng Bai^1,2,3^, Ying Lin^1,2,3^, Yufei Gou^1,2,3^, Hong Zhang^1,2,3^, Xinyue Zhang^1,2,3^, Jiayuan Wei^1,2,3^, Tianyu Chang^1,2,3^, Jingyu Li^1,2,3^, Jianli Liu^1,2,3^*

1.College of Biological Science and Engineering, North Minzu University, Yinchuan, Ningxia, China

2.Key Laboratory of Ecological Protection of Agro-pastoral Ecotones in the Yellow River Basin, National Ethnic Affairs Commission of the People’s Republic of China, Yinchuan, Ningxia, China

3.Ningxia Key Laboratory of Microbial Resources Development and Applications in Special Environment, Science and Technology Department of Ningxia, Yinchuan, Ningxia, China

*****Corresponding author: Jianli Liu

E-mail: ljl7523@126.com

**Number of tables: 1**

**Supplementary Material-****Tables**

Table S1 Relevant properties of co-occurrence networks between B_soil and Rz_soil from the three plant communities based on Spearman’s correlation coefficient (r) (|r| ≥ 0.5, *p* <0.05)

|  | Samples | Total edges | Negative edges | Positive edges | Average degree | Modularity | Density | Diameter | Average clustering coefficient | Average path distance |
| --- | --- | --- | --- | --- | --- | --- | --- | --- | --- | --- |
| Bacterial network | Tm_Rs_Sp_S_RZ_soil | 2163 | 1026 | 1137 | 10.869 | 0.506 | 0.055 | 11 | 0.288 | 2.729 |
|  | Tm_Rs_Sp_S_B_soil | 2610 | 1283 | 1327 | 13.116 | 0.487 | 0.066 | 12 | 0.306 | 3.08 |
|  | Tm_S_RZ_soil | 2095 | 904 | 1191 | 10.581 | 0.483 | 0.054 | 10 | 0.296 | 3.075 |
|  | Tm_S_B_soil | 2654 | 1257 | 1397 | 13.27 | 0.411 | 0.067 | 10 | 0.299 | 2.868 |
|  | Tm_RZ_Soil | 2490 | 1253 | 1237 | 12.45 | 0.483 | 0.063 | 9 | 0.3 | 2.85 |
|  | Tm_B_Soil | 1920 | 883 | 1037 | 9.6 | 0.586 | 0.048 | 11 | 0.289 | 3.194 |
| Fungi network | Tm_Rs_Sp_S_RZ_soil | 1123 | 460 | 663 | 5.615 | 0.674 | 0.023 | 10 | 0.328 | 3.166 |
|  | Tm_Rs_Sp_S_B_soil | 1050 | 286 | 764 | 5.25 | 0.726 | 0.026 | 11 | 0.342 | 3.501 |
|  | Tm_S_RZ_soil | 1097 | 398 | 699 | 5.485 | 0.645 | 0.028 | 10 | 0.31 | 2.836 |
|  | Tm_S_B_soil | 984 | 205 | 779 | 4.92 | 0.821 | 0.025 | 9 | 0.375 | 2.195 |
|  | Tm_RZ_Soil | 1525 | 544 | 981 | 7.702 | 0.632 | 0.039 | 11 | 0.298 | 3.79 |
|  | Tm_B_Soil | 1337 | 220 | 1117 | 6.685 | 0.772 | 0.034 | 12 | 0.398 | 3.047 |

Note: Tm_Rs_Sp_S_Rz_soil, root zone soil in plant community of *T. mongolica*, *R. songarica*, *S. passerine,* and *S. capillata*; Tm_Rs_Sp_S_B_soil, bare soil in plant community of *T. mongolica*, *R. songarica*, *S. passerine,* and *S. capillata*; Tm_S_Rz_soil, root zone soil in plant community of *T. mongolica* and *S. capillata*; Tm_S_B_soil, bare soil in plant community of *T. mongolica* and *S. capillata*; Tm_Rz_soil, root zone soil in plant community of *T. mongolica*; Tm_B_soil, bare soil in plant community of *T. mongolica*.
